# Vital Dynamics; the Hunterian Oration before the Royal College of Surgeons in London, 14th February, 1840

**Published:** 1840-10

**Authors:** 


Art. I.—*Vital Dynamics; the Hunterian Oration before the Royal College of Surgeons in London, 14th February,* 1840. By Joseph Henry Green, F.R.S., &c. 8vo, pp. 135.

"Notwithstanding the favorable testimony of friends, on whose judgment I rely," says our author, by way of preface, "to the success of the oration which I now offer to the candid and impartial criticism of those engaged in scientific pursuits, I shall neither be surprised nor offended if it should appear that some of my auditors deemed it unsuited to the occasion on which it was delivered, or that many of my readers consider it unsuited to any occasion." We have the less hesitation, therefore, in expressing our candid opinion upon the production. The following additional extract from the preface will explain the purpose of the orator: "I will not conceal from my readers that I have had an ulterior object in the following address, and, in addition to its purpose, in connexion with the attention of the founders of the Hunterian Oration, of vindicating the original merit of John Hunter as a philosophical physiologist, that it was composed with a view to the larger design, to which his labours most importantly contributed, of reconciling the study of nature with the requirements of our moral being, and of connecting science, which even as the noblest offsprings of our intellect is but a fragment of our humanity, with the philosophy of Coleridge, which, as far as my knowledge extends, preeminently, if not alone, gives life and reality to metaphysical pursuits, by showing their birth, growth, and requisite foundation in the whole man, head and heart." (p. xx.)

We are by no means inclined to deny that a clear notion of the powers of the human mind, and of the best means of bringing them to bear upon the investigation of truth, forms a most excellent preparation for the pursuit of any branch of scientific enquiry; but we think that the experience of ages has shown that between the higher branches of physics and metaphysics there is but little *practical*, however much *abstract* connexion; and that the greatest and most successful of physical philosophers have been led to their discoveries by the study of external nature only, and not by abstract meditation on the nature of ideas. We quite agree with Mr. Green that the discovery of a *law* is something very different from the mere generalization of phenomena, but any one who retraces the history of science will at once perceive that the most likely means of discovering the law is to collect and meditate on the phenomena. Did Hunter, in his search for the laws of life, sit down to the study of Plato's doctrine of ideas? Was it not rather his chief glory that he aimed to collect phenomena from every source he could devise, as materials for induction, and that he perceived that no laws could be secure that were not based on the widest possible foundation?

We cannot but think that Mr. Green's notions on the subject are themselves a little vague; they certainly appear so from the phraseology in which they are dressed. He tells us that "It was the peculiar and eminent merit of John Hunter that he had raised his mind to the apprehension of life as a law, in aid of a science of vital dynamics, and as the means of giving scientific unity to the facts of living nature." "In that very conception of a law, he taught us that life is a power anterior in the order of thought to the organization, which it animates, sustains, and repairs, a power originative and constructive of the organization in which it continues to manifest itself in all the forms and functions of animated being. This great idea never ceased to work in him as his genius and governing spirit." Now we should no more think of calling LIFE *a law*, than we should of calling physics or chemistry *laws*. They are comprehensive expressions of series of phenomena, governed by *laws*, but not as yet reducible to any *single* principle. Until it is so, we cannot see that we can think of life under any other aspect. The sense in which our author uses the term idea is also peculiar; but as he traces it to the high authority of Plato and Coleridge, we must not presume to question it. He seems to us, however, to make their abstractions yet more mystical. "That which contemplated objectively, (that is, as existing externally to the mind,)" says the last of these metaphysicians, "we call a law; the same contemplated subjectively, (that is, as existing in a subject or mind,) is an idea." Both laws and ideas, however, are represented by our author as having a *separate existence*, distinct alike from the phenomena to which they refer, and from the mind which conceives them. "We may describe an idea," says Mr. Green, (Preface, p. xxv.) "as a causative principle, combining both power and intelligence, containing, predetermining, and producing its actual result in all its manifold relations, in reference to a final purpose; and realized in a whole of parts, in which the idea, as the constitutive energy, is evolved and set forth in its unity, totality, finality, and permanent efficiency." How far such an exposition of such a doctrine was a fit subject for a Hunterian oration, our readers may judge for themselves. That the composition indicates great learning and deep thought is only saying what the attainments and talents of the author might have given reason to expect. And we should not do him justice were we not to quote some passages from it, which shall give a more favorable impression of its character than that which our readers may have derived from our previous remarks. Alluding at his commencement to the frequency with which Hunter's merits have been set forth, by which, in the opinion of some, the materials for eulogy have been exhausted, he continues:

"The discoveries of science, the triumphs of genius, the revelation of truth, seem to partake of the permanent being which is their source; they are perennial, living growths, which ever put forth anew their foliage, blossom, and golden fruit; and we collect from year to year the harvest, that owes its birth to the divine seeds of wisdom, which the gifted individuals of our race have been permitted to plant, which have been watered by the dews of heaven, and have been fostered by the light and genial warmth of a sun that sheds on them the blessing of providence." (p. 4.) Again, "If our praise is to be discriminative and appropriate to the occasion, we must essay in the humble hope of some inspiration of the same power, to scan the genius which animated and directed his bold, original, and profound researches, and to trace with kindred spirit the mind of him who conceived, planned, and in a great measure constructed (what yet indeed remains to be fully achieved) the mighty work of a philosophy and science of life and living being." (p. 5.) To our minds there is much beauty in the following passage, expressive of the pleasure derived from the contemplation of the higher laws of nature. "It is by virtue of this reason that we hear the voice and legislative words of the Creator sounding through the universe; and it is in the sabbath stillness of our intellectual being, when the busy hum of the world is hushed, that the strains of this divine music penetrate the soul attuned by meditation to move responsive to its harmony." (p. 21.)

Besides the oration, which only occupies about fifty pages of the volume, we have an Appendix containing a series of short essays, on the "Evolution of the Idea of Power," "Transcendental Anatomy," "Gradation of Animal Life," "Characteristics of Man's Bodily Frame," "Hunter's Pathology," "Instinct," and the "Recapitulatory Lecture" of the author's former Hunterian course. These will be read by many with more interest than the oration itself.

